# Hydrolysable Tannins and Biological Activities of *Meriania hernandoi* and *Meriania nobilis* (Melastomataceae)

**DOI:** 10.3390/molecules24040746

**Published:** 2019-02-19

**Authors:** Claudia Lorena Valverde Malaver, Ana Julia Colmenares Dulcey, Carlos Rial, Rosa M. Varela, José M. G. Molinillo, Francisco A. Macías, José Hipólito Isaza Martínez

**Affiliations:** 1 Department of Chemistry, Faculty of Natural and Exact Sciences, Universidad del Valle, GIPNA, Cali 760032, Colombia; claudia.valverde@correounivalle.edu.co (C.L.V.M.); ana.colmenares@correounivalle.edu.co (A.J.C.D.); 2 Allelopathy Group, Instituto de Biomoléculas (INBIO), Department of Organic Chemistry, School of Sciences, Universidad de Cadiz, C/República Saharaui 7, 11510-Puerto Real (Cadiz), Spain; carlos.rial@uca.es (C.R.); rosa.varela@uca.es (R.M.V.); chema.gonzalez@uca.es (J.M.G.M.); famacias@uca.es (F.A.M.)

**Keywords:** Melastomataceae, *Meriania*, *M. hernandoi*, *M. nobilis*, polyphenols, hydrolysable tannins, DPPH, FRAP, phytotoxicity

## Abstract

A bio-guided study of leaf extracts allowed the isolation of two new macrobicyclic hydrolysable tannins, namely merianin A (**1**) and merianin B (**2**), and oct-1-en-3-yl *β*-xylopyranosyl-(1”-6’)-*β*-glucopyranoside (**3**) from *Meriania hernandoi*, in addition to 11 known compounds reported for the first time in the *Meriania* genus. The structures were elucidated by spectroscopic analyses including one- and two-dimensional NMR techniques and mass spectrometry. The bioactivities of the compounds were determined by measuring the DPPH radical scavenging activity and by carrying out antioxidant power assays (FRAP), etiolated wheat coleoptile assays and phytotoxicity assays on the standard target species *Lycopersicum esculentum* W. (tomato). Compounds **1** and **2** exhibited the best free radical scavenging activities, with FRS_50_ values of 2.0 and 1.9 µM, respectively.

## 1. Introduction

Melastomataceae is a woody dicotyledonous family with approximately 4200 to 4500 species and 166 genera distributed mainly in the South American neotropics [1,2]. *Meriania* Swartz (Melastomataceae) is a neotropical genus of shrubs and trees with around 93 species distributed through southern Mexico, Central America, the Greater Antilles, Andean South America, the Guaiana Highlands, and south to southeastern Brazil [3]. In Colombia, 37 species of this genus have been found and these include *Meriania nobilis* Triana and *Meriania hernandoi* L. Uribe. *M. nobilis* is also called wax flower because its flowers are melliferous. *M. nobilis* is an endemic tree species in the Colombian Andes, which is its native habitat. The tree is mainly used for ornamental purposes due to its striking intense violet flowers. Similarly, *M. hernandoi* is mainly ornamental due to the intense orange color of its flowers, which is an unusual characteristic amongst the Melastomataceae family, for which violet, fuchsia and white tones prevail in flowers. These plants grow in the humid and foggy forests of southern Colombia and northern Ecuador. These two species are members of the Melastomataceae family, which is rich in phenolic constituents such as flavonoids, terpenoids, quinones, lignans and tannins [4]. These constituents are responsible for analgesic, anti-inflammatory [5], antioxidant [6], and antimicrobial [7] activities, the regeneration of gastric mucosa [8] and healing of gastric ulcer [9]; the *Meriania* genus is a small group within the Melastomataceae family, with about 71 species, 17 of which are endemic to Colombia [4]. Sites where this tree grows are very scarce and its distribution is extremely restricted. As a consequence, *Meriania* species can be easily destroyed by the advance of cultivated areas at the expense of native forest and also because they are considered useless due to a lack of ethnobotanical and phytochemical knowledge. On the basis of the information outlined above, the aim of the study reported here was to provide added value to these species and thus favour their preservation. The study involved a bio-guided evaluation of the phytochemistry of the leaves of two species, *M. nobilis* and *M. hernandoi*, as well as their antioxidant, phytotoxic and pharmacological or agroindustrial potential. Three new phytochemicals are reported, namely two new hydrolysable tannins, merianin A (**1**) and merianin B (**2**), and one alkyl glycoside (**3**), together with **11** known compounds (**4**–**14**).

## 2. Results and Discussion

### 2.1. Bio-Guided Phytochemical Study

**Figure 1:**
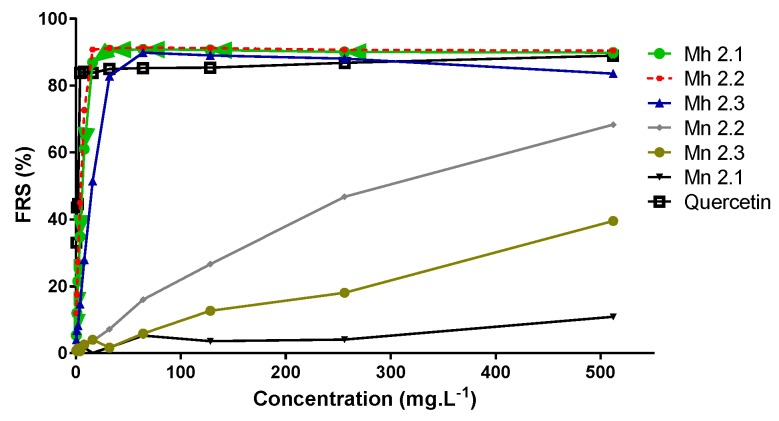
DPPH radical scavenging activity of the extracts of *M. nobilis* and *M. hernandoi*. Values are expressed as percentage DPPH radical scavenged.

The *Meriania* genus has hardly been investigated with respect to its phytochemistry or its potential biological activity. The research reported here concerned a bio-guided phytochemical study of the leaves of two species, *M. nobilis* and *M. hernandoi*. Firstly, dry leaves of *M. nobilis* and *M. hernandoi* were extracted with acetone/H_2_O 7:3. This extract was partitioned with ethyl acetate and the organic portion was denoted as Mn or Mh 2.1; the aqueous portion was partitioned with *n*-BuOH/H_2_O to obtain Mn or Mh 2.2 and Mn or Mh 2.3. The antioxidant activity and phytotoxicity of the three extracts of each species were evaluated. The antioxidant activity of the extracts was determined by measuring the DPPH radical scavenging activity, with values expressed as scavenging percentage of the DPPH radical and the ferric reducing ability (FRAP) as a measure of antioxidant power. The Total Phenolic Content (TPC) was also determined using the Folin–Ciocalteau method. The results show that the extracts Mh 2.1 and Mh 2.2 exhibited high antioxidant activity, with values of 90% and 91% scavenging of the DPPH radical, respectively (Figure 1). These extracts also showed high antioxidant power, with values 53 and 65 FeSO_4_·7H_2_O mg (100 g DE)^−1^, respectively. These values are higher than that obtained for quercetin (61 FeSO_4_·7H_2_O mg (100 g DE)^−1^) (Table 1), which was used as the reference compound. This activity also correlates with the TPC values in that the extracts that exhibited high antioxidant activity in FRAP and scavenging of the DPPH radical also had a high Total Phenolic Content. The TPC values obtained for Mh 2.1 and Mh 2.2 were 224 and 240 mg AG/g DE (Table 1), respectively. This suggests that the antioxidant activity can be attributed to the presence of phenolic compounds. In addition, the extracts obtained from *M. nobilis* exhibited moderate and low antioxidant activities, with the FRS percentage ranging from 11% to 60%, and they also showed low FRAP values (0.28–2.01 FeSO_4_·7H_2_O mg (100 g DE)^−1^) and TPC values in the range 12–26 mg AG/g DE. This finding confirmed that the antioxidant activity is associated with phenolic compounds.

**Figure 2:**
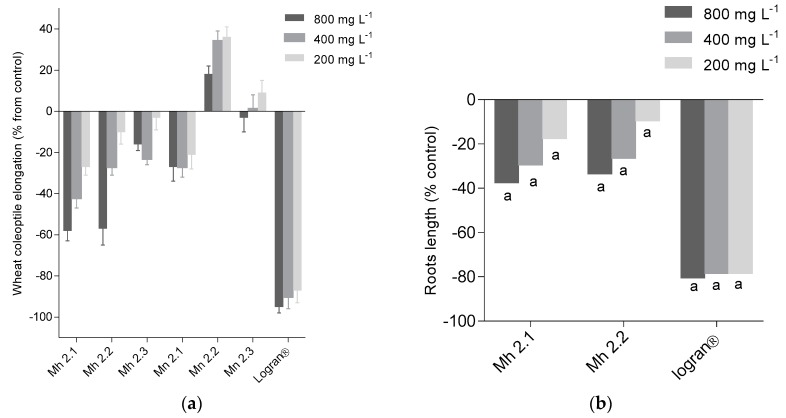
(**a**) Effect of extracts of *M. nobilis* (Mn) and *M. herndandoi* (Mh) on etiolated wheat coleoptile elongation. Values are expressed as percentage difference from the negative control. (**b**) Effect of extracts Mh 2.1 and Mh 2.2 from *M. herndandoi* on root growth of STS: *Lycopersicum esculentum* W. Values are expressed as percentage difference from the negative control and are not significantly different with *P* > 0.05 for Welch’s test. ^a^ Values significantly different with *P* < 0.01. ^b^ Values significantly different with 0.01 < *P* < 0.05.

The initial extracts were tested in a general activity bioassay, namely the etiolated wheat coleoptile assay. This is an initial screening method to evaluate activity and it is highly sensitive to pharmacological activities [10] and also shows a good correlation with phytotoxic activity. The results obtained were similar to those for antioxidant activity. The extracts Mh 2.1 and Mh 2.2 exhibited a high inhibitory effect on coleoptiles, with 58% and 57% inhibition, respectively, at the highest concentrations (Figure 2a). The extracts that showed inhibitory activity on coleoptiles were evaluated for phytotoxic activity. The assay was performed using *L. sativa* L. (lettuce), *Lycopersicum esculentum* Will. (tomato), *Lepidium sativum* L. (cress), and *Allium cepa* L. (onion) as standard target species (STS) [11]. Moreover, *Lolium perenne* and *Lolium rigidum* were used as weeds and significant effects were only observed on the root growth of STS in tomato (Figure 2b). The inhibition values for extracts Mh 2.1 and Mh 2.2 on the root growth of tomato at 800 ppm were 37% and 33%, respectively. These values are lower than those obtained with Logran® (82%), a commercial herbicide used as a positive control. According to the biological activity results, the extracts Mh 2.1, Mh 2.2 and Mn 2.2 were selected for bio-guided fractionation and isolation of the major components. 

**Figure 3:**
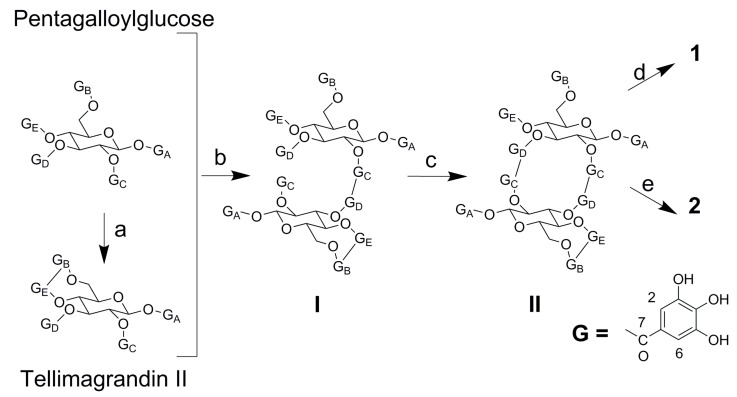
Biogenetic Approach to obtain merianin A (**1**) and merianin B (**2**), (**a**) Intramonomeric C–C oxidative coupling, (**b**,**c**) intermonomeric C–C oxidative coupling, (**d**,**e**) intramomomeric C–O–C oxidative coupling.

**Figure 4:**
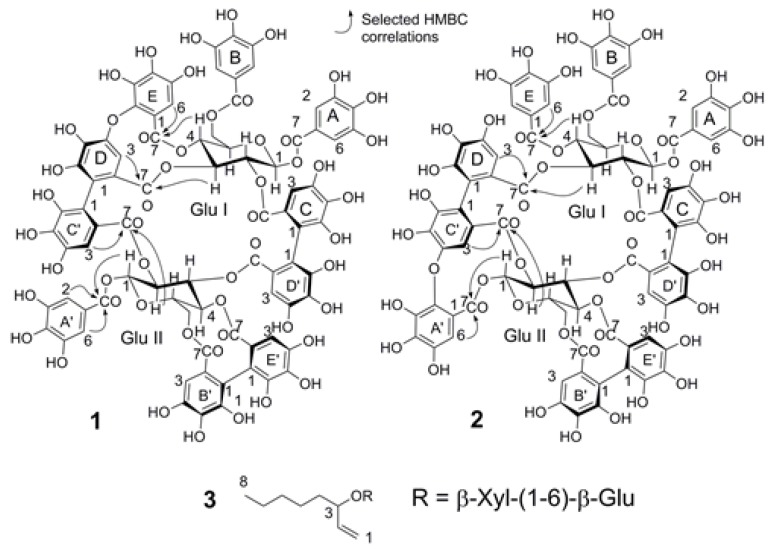
New compounds isolated from *M. hernandoi* leaves.

**Figure 5:**
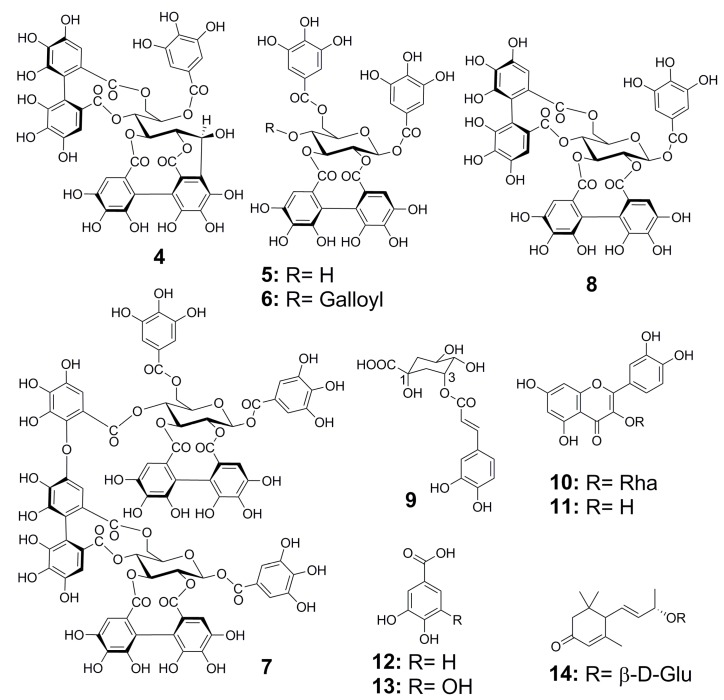
Compounds isolated from *Meriania hernandoi* and *Meriania nobilis* leaves.

In the bio-guided fractionation, the three extracts were chromatographed using DIAION HP-20 as the stationary phase and aqueous methanol as the mobile phase to obtain 11 fractions. The major compounds were isolated from the fractions that showed biological activity using MCI-Gel CHP20P as the stationary phase and aqueous methanol as the mobile phase. Three new compounds were obtained from the Mh 2.1 and Mh 2.2 extracts, namely two new isomeric hydrolysable tannins, merianin A (**1**) and merianin B (**2**), one alkyl glycoside (**3**) (Figure 3 and Figure 4), and eleven known substances (**4**–**14**): casuarinin (**4**) [12], nobotanin D (**5**) [13], pterocaryanin C (**6**) [14], nobotanin F (**7**) [13], casuarictin (**8**) [12], 3-*O*-caffeoyl quinic acid (**9**) [15], quercitrin (**10**) [16], quercetin (**11**) [17], hydroxyquinol (**12**) [18], gallic acid (**13**) (Figure 3) and vomifoliol 9-*O*-*β*-glucopyranoside (**14**) [19], which was obtained from Mn 2.2 (Figure 5).

### 2.2. Characterization of the Compounds

Compound **1** was obtained as a brown amorphous solid with a molecular weight of 1872.1738 and molecular formula C_82_H_56_O_52_, as established by the EI-MS spectrum with an ion peak at *m*/*z* 1871.1674 [M − H]^−^ and doubly charged ion at *m*/*z* 935.0801 [M − 2H]^2−^ in negative mode. The ^1^H-NMR spectrum revealed the presence of three galloyl groups by three signals that integrate to two protons each at δ 7.23, 7.09 and 6.96, which were assigned as shown in Table 2 according to long range correlations. A valoneoyl group and two hexahydroxydiphenyl (HHDP) groups were evidenced by seven 1H-singlets at δ 7.12, 6.62, 6.54, 6.48, 6.41 6.39 and 5.91, which were also assigned from the HMBC spectrum. The (*S*) configuration of both the HHDP and valoneoyl groups was confirmed by the circular dichroism (CD) spectrum, which exhibited a negative Cotton effect at around 253 nm and a positive effect near to 276 nm, i.e., similar to that of the lignan gomisin D, whose configuration is (*S*) [20], as depicted in Supplementary Materials Figure S13. The dimeric nature of **1** was demonstrated by the presence of two anomeric signals at δ_H_ 6.10 [d, *J* = 8.5 Hz, glucose (Glc) I, H-1] and 6.24 [d, *J* = 8.4 Hz, Glc II, H-1]. The other glucose proton signals were all assigned based on ^1^H-^1^H shift correlation spectroscopy (COSY) and *J*-resolved spectra, which indicated that the conformation adopted by the two glucoses is ^4^C_1_ and that they are fully acylated. A large difference between the chemical shifts of the H-6 proton signals on Glc II (∆H6 = 1.45) suggested the presence of a biphenyl moiety with bridged ester linkages between *O*-4/*O*-6 [21] as in telimagrandin II [22]. The location of this unit on the glucose core and its exact attachment mode were determined based on the HMBC three-bond correlations from the corresponding protons of the glucose units and from the aromatic protons to the carbonyl carbons, as summarized in Table 3 and Figure 3. The signal at δ 7.12 (valoneoyl E-6) showed connectivity to the glucose I H-4 (5.92) at three bonds with an ester carbonyl at 165.7 ppm. Similarly, the signal at δ 5.91 (valoneoyl C’-3) showed coupling to the glucose II H-2 (5.09) and to an ester carbonyl at 169.5 ppm and the signal at δ 6.48 (valoneoyl D-3) correlated to the glucose I H-3 (5.54) and with an ester carbonyl at δ 170.5. These spectroscopic data for 1 were different to those of the isomer nobotanin B [23], especially for the valoneoyl signal at 5.91 ppm in 1, which is shifted downfield with respect to the signal at 6.12 ppm in nobotanin B, and also the sugar signals H-3, H-4 and H-6. The evidence outlined above is consistent with compound 1 being a dimer of pentagalloyl glucose [24] and tellimagrandin II; which, in a biogenetic approach, is initially linked by oxidative C–C coupling between the galloyl units on C-2 of the pentagalloyl glucose and that on C-3 of the tellimagandin II to give intermediate I (Figure 3). In the next step, the macrocycle is generated by another oxidative coupling between the galloyl units from C-2 of telimagrandin II to C-3 of pentagalloyl glucose to generate intermediate II. An oxidative C–O–C coupling between rings D and E of positions 3 and 4 of pentagalloyl glucose leads to the formation of merianin A (1); while the oxidative C–O–C coupling between the 1 and 2 of telimagrandin II produces the merianin B isomer (**2**). The letters of the aromatic rings were assigned according to the sequence of galloylation in the biosynthesis of the pentagalloyl glucose. This biogenetic proposal is reinforced by the downfield shift of carbons C-2 and C-3 in both glucose units, with respect to pentagalloyl glucose and tellimagrandin II, as shown in Table 3. Acid hydrolysis followed by methylation and silylation of 1 confirmed the identity of the sugars in the molecule as d-glucose. The structure of compound **1** was therefore established as the macrobicyclic hydrolysable tannin merianin A (**1**), as shown in Figure 4.

Compound **2** was obtained as a brown amorphous powder and had a molecular weight of 1872.1689 and molecular formula C_82_H_56_O_52_, as established by the HREI-MS doubly charged ion peak at *m*/*z* 935.0765 [M − 2H]^2−^ in negative mode. The (*S*) configuration of the HHDP and valoneoyl groups was confirmed by the circular dichroism (CD) spectrum, which exhibited a negative Cotton effect at around 253 nm and positive effect close to 276 nm, as for compound **1**, (see Supplementary Materials Figure S14). The ^1^H-NMR and ^13^C-NMR spectra were virtually identical to those obtained for 1. A subtle difference between 2 and 1 was evidenced by the HMBC correlation. The three-bond correlation from proton signals at δ 7.12 (valoneoyl A’-6) and glucose II H-1 (6.24) to the ester carbonyl at δ 165.7 provided evidence for the C–O–C oxidative coupling between the galloyl moieties on C-1 and C-2 of telimagrandin II monomeric unit (Figure 3 and Figure 4). Furthermore, the downfield shifts of C-2 and C-3 with respect to the corresponding signals of telimagrandin II, as shown in Table 3, support the presence of the large macrocycle. Therefore, the signals at δ 6.96 (galloyl E) and the glucose I H-4 (5.92) exhibited three-bond coupling to the ester carbonyl at δ 165.7 (Figure 3). Acid hydrolysis followed by methylation and silylation of 2 confirmed the identity of the sugars as d-glucose. Consequently **2** is an isomer of **1** and it was named merianin B.

Compound **3** was obtained as a brown syrup and it had a molecular weight 422.2147 and molecular formula C_19_H_34_O_10_, as established by the HREI-MS ion peak at *m*/*z* 421.2067 [M − H]^−^ in negative mode. The ^1^H-NMR spectrum revealed two anomeric protons at δ 4.35 (d, *J* = 7.9) and 4.34 (d, *J* = 7.8). The other sugar proton signals were all assigned based on ^1^H-^1^H shift correlation spectroscopy (COSY), total correlated spectroscopy (TOCSY) and *J*-resolved spectroscopy (*J*-res), which indicated a sequential *trans* diaxial relationship between H-1’ (4.35) and H-5’ (*J* = 7–9 Hz) corresponding to *β*-glucopyranose and a sequential *trans* diaxial relationship between H-1” (4.34) and H-4” (*J* = 7–9 Hz) indicated a *β*-xylopyranose (Table 4). The spectra contained signals that evidence a linear structure of an octenyl moiety, with three signals at 5.05 (dd, *J* = 3.7, 10.5), 5.18 (dd, *J* = 3.7, 17.3) and 5.82 (ddd, *J* = 7.1, 10.5, 17.3) (Table 4) indicating the presence a double bond in the molecule. The linkage between the aglycon and sugar moiety was determined based on the HMBC correlations, which showed coupling of glucose H-1’ and C-3 to three bonds, with evidence of coupling for H-6’ with C-1”to three bonds indicating interglycosidic linkage. Silylation of 3 confirmed the identity of the sugars in the molecule. The spectroscopic evidence is consistent with this compound being oct-1-en-3-yl *β*-xylopyranosyl-(1”-6’)-*β*-glucopyranoside (**3**) (Figure 4). A similar compound has been isolated from the Melastomataceae family and this was designated as a matsutake alcohol derivative [25].

### 2.3. Bioactivity of Compounds

The antioxidant activities of the isolated compounds were determined using the DPPH assay and values are expressed as the concentration of the sample required to scavenge 50% of the DPPH free radicals (FRS_50_) (Table 1). The results show that all of the compounds isolated from *M. hernandoi* exhibited low FRS_50_ values and these are comparable to the value obtained for quercetin. These compounds possess a high scavenging capacity for free radicals. The hydrolysable tannins showed the highest scavenging capacity for free radicals and this is due to the fact that a hydrolysable tannin can donate a hydrogen atom and form a stable quinone [26,27]. In addition, compounds **1** and **2** exhibited the best free radical scavenging activity, with FRS_50_ values of 2.0 and 1.9 µM, respectively, since these compounds have a dimeric structure with more catechol-type hydroxyl groups.

The results obtained for the antioxidant power (FRAP) show that all of the compounds isolated from *M. hernandoi* have high values. This finding correlates with the FRS_50_ results. The hydrolysable tannins tested had a high antioxidant power—especially the dimeric tannins. In the FRAP assay, the activity values for phenolic compounds seem to depend on the degree of hydroxylation and the extent of conjugation of the phenolic compounds [28]. For example, compounds **1** and **2**, which are macrocyclic, and **7**, which has a dimeric structure, presented the highest FRAP values (Table 1).

**Figure 6:**
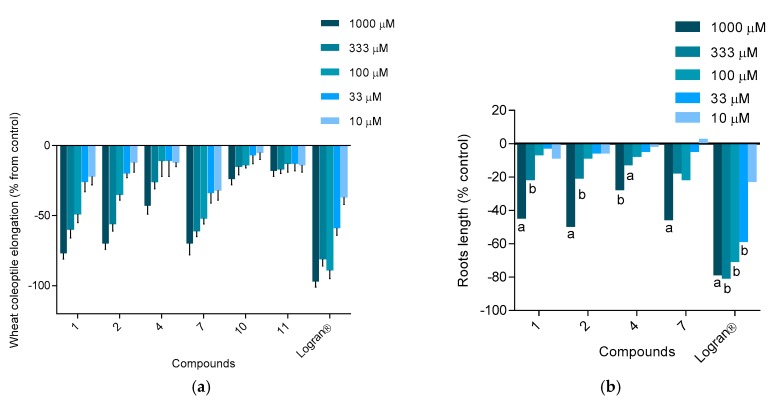
(**a**) Effects of compounds **1**, **2**, **4**, **7**, **10** and **11** on etiolated wheat coleoptile elongation. Values are expressed as percentage difference from the negative control. (**b**) Effects of compounds **1**, **2**, **4** and **7** on root growth of STS: *Lycopersicum esculentum* W. Values are expressed as percentage difference from the negative control and are not significantly different with *P* > 0.05 for Welch’s test. ^a^ Values significantly different with *P* < 0.01. ^b^ Values significantly different with 0.01 < *P* < 0.05.

Compounds **1**, **2**, **4**, **7**, **10** and **11** were available in larger quantities and these were tested in the etiolated wheat coleoptile assay. The values obtained were high for the macrocyclic tannins and the dimeric hydrolysable tannin, with values of 77% for merianin A (**1**), 70% for merianin B (**2**) and 70% for nobotanin F (**7**) at a concentration of 1000 µM. Casuarinin (**4**) is a monomeric tannin and this showed only moderate activity, with a value of 43% at 1000 µM. In contrast, the compounds quercitrin (**10**) and quercetin (**11**) showed low activity, with values of 24% and 18% (Figure 6), respectively. Similar results were obtained in the phytotoxicity assay; the strongest inhibition was observed for merianin A (**1**), merianin B (**2**) and nobotanin F (**7**), with values in the range 40–50% at a concentration of 1000 µM. The monomeric tannin casuarinin (**4**) showed low phytotoxicity, with a value of only 27% at 1000 µM (Figure 4b). Significant effects were only observed on the root growth in *Lycopersicum esculentum* Will (tomato). The results for the inhibition of coleoptile growth and phytotoxicity show that activity increases with the molecular size of the tannin, as the compounds with a dimeric structure or macrocycles showed higher activity than the monomer casuarinin. Likewise, the activity seems to be related to the number of galloyl groups and HHDP groups in the molecule. This trend has also been reported for other types of activity, such as cytotoxic activity against human tumor cell lines [29] and neuraminidase inhibitory activity in anti-influence therapy [30], where the activity is enhanced by increasing the number of free galloyl groups or HHDP. A total of 14 compounds have been isolated from *M. hernandoi* and *M. nobilis* and three of these are described for the first time. Among the major compounds, the new hydrolysable tannins **1** and **2**, along with compound **7**, show higher activities in the etiolated wheat coleoptile bioassay and against the STS tomato seeds. Furthermore, together with the antioxidant activities, compound **4** showed different biological activities such as anti-herpes [31], neuroprotective effects by suppressing glutamate-mediated apoptosis in human cells [32] and therapeutic properties against inflammatory skin diseases [33]. Compound **8** also proved to be an α-glucosidase inhibitor [34]. The results obtained show that the species *M. hernandoi* is a good source of promising bioactive metabolites that include pharmacological activities.

## 3. Materials and Methods 

### 3.1. General Experimental Procedures 

Optical rotations were measured with a JASCO (Tokyo, Japan) model P-2000 series digital polarimeter. UV-visible spectra were recorded using a JASCO (Tokyo, Japan) UV-730 spectrophotometer. ^1^H and ^13^C-NMR spectra were recorded in methanol-*d*_4_ or acetone-*d*_6_ + D_2_O with a Bruker (Bruker-Biospin GmbH, Rheinstetten, Germany) Advance III 400 spectrometer at 400 MHz and 100 MHz and Agilent Technologies (Santa Clara, CA, USA) spectrometers at 500 MHz and 125 MHz or at 600 MHz and 200 MHz. A combination of 1D spectra such as ^1^H-NMR, ^13^C-NMR, DEPT135, and 2D spectra such as ^1^H-^1^H-COSY, ^1^H-^1^H-TOCSY, ROESY, *J*-resolved, HSQC and HMBC were used to determine the chemical structures. A selection of these spectra is provided in the Supplementary File.

HPLC analysis was performed on a Shimadzu Corp. LC-2010 instrument (Kyoto, Japan), equipped with a CBM-20 A system controller, a DGU-20 A degasser, a LC-20AD pump, a CTO-20AC column oven, an SIL-20AHT autosampler and an SPD-M20A PDA detector. Analytical HPLC was performed on a kinetex phenomenex column (75 mm length - 4.6 mm ID - 2.6 µm), using a mobile phase of 0.1% formic acid (A) and methanol (B), and detection was carried out at 200–400 nm. The following gradients of mobile phase were used at a flow rate of 1 mL/ min: 5% B, 0 min; 5–70% B, 15 min; 70% B, 17.5 min; 70–5% B, 19 min; 5% B, 20 min. Semipreparative HPLC was performed on a Restek Pinnacle II C18 5 µm (250 mm length - 21.2 mm ID). Exact masses were measured on a UPLC-QTOF ESI (Waters Synapt G2, Manchester, UK) high-resolution mass spectrometer (HRTOFESIMS). Mass spectra were recorded in the negative- or positive-ion mode in the range *m*/*z* 100–2000, with a mass resolution of 20,000 and an acceleration voltage of 0.7 kV. CD spectra were measured with a J-1500 Circular dichroism Spectrophotometer. GC/MS analysis of the trimethylsilylated derivatives was performed on a Shimadzu model GCMS QP2010 Ultra system, operated in electronic ionization (EI) mode at 70 eV in full scan mode (range 35–700 *m*/*z*) with a scan speed of 1428 scans s^−1^. Separation of the methylsilylated and silylated monosaccharides was performed on a Restek (Bellefont, PA, USA) chemically bonded Rtx-5MS fused-silica capillary column (30 m × 0.25 mm i.d. × 0.25 μm film thickness) with temperature program as follows: 150 °C for 2 min, ramp to 250 °C at 3 °C min^−1^, and hold at 250 °C for 3 min. The injector was operated at 280 °C in split mode at a 1:10 split ratio. Helium with a purity of a 99.999% was used as the carrier gas at a flow rate of 1.0 mL min^−1^, Flow Control by linear velocity at 38.0 cm/s. Samples (1.0 μL) were injected with an AOC- 20i+s autosampler, 10 μL Hamilton micro syringe (Reno, NV, USA).

### 3.2. Plant Material

*Meriania nobilis* Triana (Voucher CUVC-51735) was collected at Caldas, Antioquia, Colombia during May 2012 and *Meriania herandoi* L. Uribe (Voucher CUVC-62003) at “Reserva Civil el Refugio” in Dagua, Valle del Cauca, Colombia during November 2013. Both species, in the flowering stage, were identified in the herbarium “Luis Sigifredo Espinal” at the “Universidad del Valle”.

### 3.3. Extraction and Isolation

Dried and powdered leaves (500 g) of each species were successively extracted three times with *n*-hexane followed by acetone/water 70% in a Branson Scientific ultrasonic bath at room temperature for 15 minutes. The solvent was evaporated under reduced pressure. The extract was liquid-liquid partitioned in ethyl acetate to obtain extracts Mn 2.1 (5.14 g, 1.1%) and Mh 2.1 (8.1 g, 1.6%) and in water-saturated *n*-BuOH to obtain extracts Mn 2.2 (2.01 g, 0.4%) and Mh 2.2 (28.3 g, 5.7%) and aqueous fractions were denoted as Mn 2.3 (13.6 g, 2.7%) and Mh 2.3 (15.1 g, 3.0%). The extracts Mn 2.1, Mh 2.1 and Mh 2.2 were fractionated using a water/MeOH stepwise gradient (10–100% MeOH, increment of 10% in each step) on a DIAION HP-20 (30 cm × 5 cm I.D) to obtain eleven fractions. The fractions that exhibited activity were chromatographed on an MCI-Gel CHP20P (460 mm length × 26 mm I.D) with a water/MeOH gradient (10–100% MeOH, increments of 5% in each step) to obtain eleven pure compounds from *M. hernandoi* and one pure compound from *M. nobilis*. The compounds were characterized as follows:

#### 3.3.1. Merianin A (**1**) 

Brown amorphous powder; ${\left[\alpha \right]}_{D}^{25}$ 86.3° (4.0 × 10^−2^, MeOH); HR-ESIMS: *m*/*z* 1871.1674 [M − H]^−^ and *m*/*z* 935.0801 [M − 2H]^2−^; UV *λ_max_* (MeOH) nm log (ε) 273 (8.5), 218 (8.8); CD (MeOH) [θ] (nm): 31.1 × 10^4^ (235), 19.5 × 10^4^ (246), −86.5 × 10^3^ (268), 23.7 × 10^3^ (279), −29.4 × 10^3^ (312). ^1^H-NMR (500 MHz, Methanol *d*_4_) δ ppm: see Table 2 and Table 3. ^13^C-NMR δ 170.7 (D-7), 170.5 (D’-7), 170.0 (C-3), 169.6 (B’-7), 169.5 (C’-7), 169.1 (E’-7’), 168.0 (A’-7), 165.9 (B-7), 165.7 (2C, A-7, E-7), 147.6 (D-4), 146.6 (B-3,5), 146.4 (2C, A, A’-3,5), 146.0 (D’-4), 145.9 (D-4), 145.8 (3C, C-4, B’-4, C’-4), 145.5 (E-6), 145.2 (D’-6), 145.0 (B’-6), 144.9 (2C, C-6, C’-6), 144.8 (D-6), 144.0 (E-5), 141.7 (E-4), 140.8 (E-3), 140.7 (B-4), 140.6 (A-4), 139.9 (A’-4), 137.5 (C’-5), 137.5 (2C, B’-5, D-5), 137.4 (C-5), 137.1 (D’-3), 136.9 (D-5), 136.5 (E-2), 126.2 (2C, B’-2, D’-2), 126.1 (2C, D-2, C’-2), 125.9 (C’-2), 125.7 (C-2), 121.2 (A’-2), 119.8 (A-2), 119.6 (B-2), 117.3 (D-1), 116.7 (B’-1), 116.5 (C’-1), 115.3 (2C, C-1, D-1), 114.6 (D’-1), 114.1 (E-1), 110.5 (4C, A-2,6, B-2,6), 110.4 (A’-2,6), 108.5 (B’-3), 108.0 (C’-3), 107.8 (D-3), 107.5 (C-3), 107.3 (D’-3), 103.5 (D-3), for sugar moieties, see Table 2. Methyl 2,3,4,6-tetrakis-*O*-(trimethylsilyl)-*α*/*β*-d-glucopyranoside. *t*_R_ 9.883/9.590, RI 1772/1756, MS *m*/*z* (%): 73 (100), 204 (60.89/69.78), 217 (13.02/11.58). HPLC *t*_R_ 5.599 min.

#### 3.3.2. Merianin B (**2**)

Brown amorphous powder; ${\left[\alpha \right]}_{D}^{25}$ 159.0 (1.3 × 10^−2^, MeOH). ESIM: *m*/*z* 935.0765 [M − 2H]^2−^; UV *λ_max_* (MeOH) nm log (ε) 271 (9.6) 218 (9.92); CD (MeOH) [θ] (nm): 41.9 × 10^3^ (235), 25.8 × 10^3^ (246), −12.1 × 10^3^ (268), 4.4 × 10^3^ (279), −4.3 × 10^3^ (312). ^13^C-NMR δ 169.2 (D-7), 169.1 (D’-7), 168.6 (C-3), 168.2 (B’-7), 168.1 (C’-7), 167.7 (E’-7’), 166.6 (A-7), 164.5 (B-7), 164.3 (2C, A’-7, E-7), 146.2 (C’-4), 145.2 (B-2,6), 145.0 (2C, A, E-3,5), 145.0 (A’-4), 144.6 (D-4), 144.5 (C-4), 144.4 (3C, D-4, B’-4, E’-4), 144.1 (C’-6), 143.8 (D’-6), 143.6 (D-6), 143.5 (2C, C-6, B’-6), 143.4 (E’-6), 142.6 (A’-5), 140.3 (A’-4), 139.4 (E-4), 139.3 (A’-3), 139.1 (B-4), 138.5 (A-4), 136.1 (3C, D-5, C-5, E’-5), 136.0 (B’-5), 135.7 (C-5), 135.5 (C’-5), 135.1 (A’-2), 124.8 (E’-2), 124.7(C-2), 124.7 (2C, B’-2, D-2), 124.5 (D’-2), 124.3 (C’-2), 119.8 (A-1), 119.0 (A-1), 118.4 (B-2), 118.2 (E-2), 115.9 (C’-1), 115.3 (B’-1), 115.1 (E’-1), 113.9 (2C, D’-1, C-1), 113.2 (D’-1), 112.6 (A’-1), 109.1 (A’-6), 109.0 (A, B, E-2,6), 107.1 (D’-3), 106.6 (C-3), 106.4 (B’-3), 106.1 (E’-3), 105.8 (D-3), 102.1 (C’-3), for sugar moieties, see Table 2. Methyl 2,3,4,6-tetrakis-O-(trimethylsilyl)-*α*/*β*-d-glucopyranoside. *t_R_* 9.883/9.590, RI 1772/1756, MS *m*/*z* (%): 73 (100), 204 (60.89/69.78), 217 (13.02/11.58). HPLC *t*_R_ 6.779 min 

#### 3.3.3. Oct-1-en-3-yl β-xylopyranosyl-(1”-6’)-β-glucopyranoside (**3**)

Brown syrup; ${\left[\alpha \right]}_{D}^{25}$ 45 (4.5 × 10^−2^, MeOH); ^1^H-NMR (400 MHz, Acetone *d*_6_ + D_2_O) δ ppm: 5.82 (1H, ddd, *J* = 7.1, 10.5, 17.3, H-2), 5.18 (1H, dd, *J* = 3.6, 17.3, H-1a), 5.05 (1H, dd, *J* = 3.6, 10.5, H-1b), 4.35 (1H, d, *J* = 7.8, H-1’), 4.34 (1H, d, *J* = 7.6, H-1’’), 4.09 (1H, q, *J* = 5.6, 8.7, H-3), 3.98 (1H, dd, *J* = 2.1, 11.5, H-5b’’), 3.81 (1H, dd, *J* = 5.3, 11.4, H-6b’), 3.70 (1H, dd, *J* = 4.8, 11.5, H-5a’’), 3.49 (1H, dd, *J* = 4.8, 8.5, H-4’’), 3.43 (1H, dd, *J* = 8.1, 10.4, H-5’), 3.41 (1H, dd, *J* = 6.5, 9.1, H-3’), 3.39 (1H, dd, *J* = 6.5, 8.1, H-4’), 3.36 (1H, ft, *J* = 8.8, H-3’’), 3.21 (1H, dd, *J* = 7.8, 9.1, H-2’), 3.21 (1H, dd, *J* = 7.6, 9.1, H-2’’), 3.18 (1H, dd, *J* = 10.1, 11.4, H-6a’), 1.60 ( 1H, dd, *J* = 10, 13.6, H-4b), 1.45 (1H, d, *J* = 8.7, H-4a), 1.30 (2H, ft, *J* = 11, H-7), 1.22 (2H, d, *J* = 11, H-5), 1.22 (2H, d, *J* = 11,H-6), 0.8 (3H, ft, *J* = 7.2, H-8). ^13^C-NMR δ 140.2 (C-2), 116.1 (C-1), 102.5 (2C, C-1’, C-1’’), 82.1 (C-3), 77.1 (C-3’), 76.6 (C-4’), 75.8 (C-3’’), 74.3 (C-2’’), 73.8 (C-2’), 70.2 (C-4’’), 70.1 (C-5’), 68.7 (C-6’’), 65.9( C-6’), 34.9 (C-4), 32.2 (C-5), 24.9 (C-6), 22.9 (C-7), 14.1 (C-8). 1,2, 3, 4-tetrakis-O-(trimethylsilyl)- *α*/*β*-d-xylopyranoside. *tR* 34343/36.886, RI: 1755/1875, MS *m*/*z* (%): 73 (100).

### 3.4. Methanolysis and Silylation

Compounds **1**, **2** and authentic monosaccharides (Sigma-Aldrich, Saint Louis, MI, USA), including d-(−)-arabinose, d-(−)-lyxose, d-(−)-fructose, l-(−)-fucose, l-rhamnose, d-(+)-xylose, d-(−)-ribose, d-(+)-mannose, d-(+)-galactose and d-(+)-glucose (1 mg), were heated with 2 N methanolic HCl (0.5 mL) for 3 h in a boiling water bath to give 1-*O*-methyl glycosides. After removal of the solvent by evaporation in vacuo, 1 mL of acetonitrile was added and free hydroxyl groups were trimethylsilylated with 100 μL of *N*,*O*-bis(trimethylsilyl)trifluoroacetamide (BSTFA) and trimethylchlorosilane (TMCS) (99:1) (Supelco) for 15 min at 70 °C.

### 3.5. Measurement of DPPH Radical Scavenging Activity 

DPPH free radical scavenging was determined using a 96-well microplate format based on the method of Sdiri and co-workers [35] with slight modification. The samples (extract, fraction, positive control or pure compounds) were prepared at 1024 mg/L in methanol. The sample (100 mL) at two-fold serial dilutions (1–512 mg/L) was added to the microplate and mixed with 100 mL of DPPH solution (132 mg/L) in methanol. After 1 h of reaction at room temperature, the absorbance of the mixture was measured at 520 nm using a microplate reader (Metertech (Taipei, Taiwan), AccuReader M 965+). Quercetin was used as the positive control and experiments on quercetin were performed in parallel. All samples were measured in triplicate. The results are expressed as percentage of radical scavenging activity (%FRS) according to the following equation:

%FRS = (A_C_ − A_S_)/Ac × 100

where A_C_ is the absorbance of the DPPH radicals without the sample or positive control and as is the absorbance of DPPH radicals with sample or positive control. The efficient concentrations of the samples and positive controls that inhibit FRS_50_ were calculated and are expressed as mg/L.

### 3.6. Measurement of Ferric Reducing Antioxidant Power (FRAP)

The reducing power from Fe^3+^ to Fe^2+^ by an antioxidant was determined using the method of Benzie and Strain [36] adjusting for use in a 96-well microplate. The extracts and the standard were prepared at 1024 mg/L, the pure compounds and standard were prepared at 250 µM in methanol or water. The FRAP reagent was prepared by mixing acetate buffer (300 mM, pH 3.6), 10 mM TPTZ (2,4,6-tripyridyl-s-triazine) in 40 mM acidified methanol, and 20 mM FeCl_3._ 6H_2_O at a 10:1:1 ratio (*v*/*v*/*v*) in this order. The mixture was then heated for 1 h at 37 °C and allowed to stand at room temperature. Immediately, two-fold serial dilutions (1–512 mg/L to extracts and 15.6–250 µM to pure compounds) of the sample (30 mL) were mixed with 140 mL of FRAP reagent and 30 mL of water on the microplate. Finally, the absorbance was measured at 600 nm in a microplate reader (Metertech, AccuReader M 965+) after 1 h of reaction. Quercetin was used as the positive control and was examined in parallel experiments. All samples were measured in triplicate. A standard calibration curve for iron(II) sulfate heptahydrate (FeSO_4_.7H_2_O) was plotted. All results are expressed as mg FeSO_4_.7H_2_O (100 g)^−1^ dry extract and as µM FeSO_4_.7H_2_O 

### 3.7. Total Phenolic Content Measured Using the Folin–Ciocalteu Method (TPC)

Total phenolic content was measured using the Folin–Ciocalteu method described by Sdiri and co-workers [35]. This method was applied in the 96-well microplate format. The samples (extract, fraction or standard) were prepared at 1024 mg/L in 2-propanol or methanol. The sample (100 mL) at two-fold serial dilutions (512–15.6 mg/L) was mixed with 50 mL of 20% *v*/*v* Folin–Ciocalteu reagent and 50 mL of sodium carbonate solution (1.6% *w*/*v*) on the microplate. The mixture was heated for 1 h at 60 °C and then allowed to cool down to room temperature. The absorbance was measured at 650 nm in a microplate reader (Metertech, AccuReader M 965+). The samples were measured in quadruplicate. Gallic acid (GA) (1–512 mg/L) was used as the standard in the calibration curve, with the optimal range of 32 to 1.0 mg/L. Average results are expressed as mg GA (100 g)^−1^ dry extract.

### 3.8. Etiolated Wheat Coleoptile Assays

All extracts were tested in this general activity bioassay and this initial screening was employed to evaluate activity. Wheat seeds (*Triticum aestivum* L.) were used in this bioassay according to the methodology previously described in the literature [35]. The extracts were prepared at 800, 400 and 200 mg L^−1^ and the pure compounds at 1000, 333, 100, 33 and 10 µM. A buffered nutritive aqueous solution with DMSO (0.5% *v*/*v*) without any tested extract was used as negative control. The commercial herbicide Logran® was used as positive control. Coleoptile elongation was measured using the Photomed© system. The results are presented in bar charts and are shown as percentage differences from the control. Thus, zero represents the control, negative values represent inhibition and positive values denote stimulation of the evaluated parameter.

### 3.9. Standard Target Species Bioassay (STS) and Weeds

The extracts that exhibited activity in the general bioassay were tested in this bioassay. *L. sativa* L. (lettuce), *Lycopersicum esculentum* Will. (tomato), *Lepidium sativum* L. (cress), and *Allium cepa* L. (onion) were used as standard target species (STS). The weeds *Lolium perenne* and *Lolium rigidum* were also used and this bioassay was carried out according to the methodology previously described in the literature [37]. The samples were prepared with aqueous buffer solution at 10^−2^ M of 2-[*N*-morpholino]ethanesulfonic acid (MES) and 1 M NaOH and were dissolved in DMSO to obtain working concentrations of 800, 400 and 200 mg L^−1^ for extracts and 1000, 333, 100, 33 and 10 µM for pure compounds. Each sample contained a constant quantity of 0.5% DMSO. The negative controls were the buffer containing DMSO without the tested compounds and the positive control was Logran®. The measurements (shoot and root length, and germination) were made using a Fitomed© system, which allowed automatic data acquisition and statistical analysis with the associated software. Results are presented as percentage differences from the control. Zero represents control, positive values represent stimulation, and negative values represent inhibition.

## Figures and Tables

**Table 1:** Antioxidant activity of extracts and compounds from the aerial parts of *M. nobilis* and *M. hernandoi*.

	FRS_50_ (μM) ^a^	FRAP (mg Fe^2+^/100 g DE)	TPC (mg AG/g DE) ^b^	FRAP (µM Fe^2+^)
Mn 2.1		0.28 ± 0.01	12 ± 0.1	
Mn 2.2		2.01 ± 0.08	26 ± 2	
Mn 2.3		0.57 ± 0.03	24 ± 1	
Mh 2.1		53.2 ± 1.5	224 ± 7	
Mh 2.2		64.5 ± 1.5	240 ± 9	
Mh 2.3		7.45 ± 0.20	120 ± 3	
Quercetin	14 ± 1	60.8 ± 1.3		337 ± 1
1	2.0 ± 0.3			366 ± 5
2	1.9 ± 0.1			364 ± 5
3	19 ± 2			168 ± 1
4	3.3 ± 0.6			274 ± 1
5	7.5 ± 0.1			268 ± 1
6	^c^			^c^
7	4.1 ± 0.1			352 ± 6
8	^c^			^c^
9	19 ± 1			252 ± 2
11	19 ± 1			263 ± 1
12	48 ± 1			166 ± 3
13	19 ± 1			283 ± 3
14	473 ± 3			44 ± 1

Data are expressed as the mean ± SD. ^a^ Concentration of the sample required to scavenge 50% of the DPPH free radicals. ^b^ Total phenolic content measured using the Folin–Ciocalteu method. ^c^ Not tested.

**Table 2:** ^13^C and ^1^H-NMR data of merianin A (**1**) and merianin B (**2**)**.**

	1 ^a^		2 ^b^		Tellimagrandin II [22]		1,2,3,4,6-pentagalloyl-*O*-*β*-glucose [24]	
Glc I	^1^H	^13^C	^1^H	^13^C	^1^H	^13^C	^1^H	^13^C
1	6.10 d (*J* = 8.5)	92.7	6.09 d (*J* = 8.4)	91.2			6.39 d (*J* = 9.5)	93.4
2	5.23 dd (*J* = 8.5, 10)	76.1	5.22 dd (*J* = 8.4, 10)	76.2			5.66 t (*J* = 9.5)	71.9
3	5.54 ft (*J* = 9.6)	78.4	5.53 dd (*J* = 8.4, 10.7)	77			6.06 t (*J* = 9.5)	73.5
4	5.92 ft (*J* = 9.6)	67.5	5.89 ft (*J* = 8.9)	68.5			5.70 t (*J* = 9.5)	69.5
5	3.44 d (*J* = 9.6)	74.3	3.43 d (*J* = 8.9)	74.7			4.60 m	74.1
6	3.79 dd (*J* = 2.7, 13.5)	64.0	3.77 d (*J* = 11)	62.6			4.60 m	62.9
	4.77 d (*J* = 13.5)		4.76 d (*J* = 11)				4.45 dd (*J* = 3, 12)	
Glc II								
1	6.24 d (*J* = 8.7)	92.7	6.24 d (*J* = 8.4)	91.3	6.20 d (*J* = 8.0)	93.8		
2	5.09 ft (*J* = 8.7)	77.0	5.08 ft (*J* = 9.8)	75.5	5.58 dd (*J* = 8.0, 9.5)	71.8		
3	5.73 dd (*J* = 8.7, 10)	77.6	5.73 ft (*J* = 9.8)	76.2	5.83 t (*J* = 9.5)	73.3		
4	5.16 ft (*J* = 10)	69.9	5.15 ft (*J* = 9.8)	68.5	5.20 t (*J* = 9.5)	70.8		
5	4.58 dd (*J* = 6.9, 10)	73.8	4.57 dd (*J* = 6.4, 13.5)	72.4	4.54 dd (*J* = 6.0, 9.5)	73.1		
6	3.93 dd (*J* = 2, 13.6)	63.9	3.92 d (*J* = 11.6)	62.4	5.36 dd (*J* = 6.0, 13)	63.1		
	5.38 dd (*J* = 6.9, 13.6)		5.37 dd (*J* = 6.4, 11.6)		3.87 d (*J* = 13)			

Methanol *d*_4_. ^a 1^H-NMR 500 MHz, ^13^C-NMR 126 MHz, ^b 1^H-NMR 600 MHz, ^13^C-NMR 200 MHz.

**Table 3:** Connectivity between aromatic and glucose protons through the ester carbonyl carbon (three-bond coupling) for merianin A (**1**) and merianin B (**2**).

	1 ^a^			2 ^b^	
Aromatic-H	-COO-	Glucose-H	Aromatic- H ^b^	-COO-	Glucose-H
Galloyl			Galloyl		
6.96 A-2,6	165.7	6.24 Glc II (H-1)	7.22 A-2,6	166.6	5.37 Glc I (H-6)
7.09 B-2,6	165.9	6.10 Glc I (H-1)	7.07 B-2,6	164.5	6.09 Glc I (H-1)
7.23 A’-2,6	168.0	5.38 Glc I (H-6)			
			7.11 E-2,6	164.3	5.89 Glc I (H-4)
HHDP			HHDP		
6.39 C-3	170.0	5.23 Glc I (H-2)	6.38 C-3	168.6	5.22 Glc I (H-2)
6.41 D’-3	170.5	5.73 Glc II (H-3)	6.40 D’-3	169.1	5.73 Glc II (H-3)
6.54 E’-3	169.1	5.16 Glc II (H-4)	6.53 E’-3	167.7	5.15 Glc II (H-4)
6.62 B’-3	169.6	3.93 Glc II (H-6)	6.60 B’-3	168.2	3.92 Glc II (H-6)
Valoneoyl			Valoneoyl		
6.48 D	170.7	5.54 Glc I (H-3)	6.47 D	169.2	5.53 Glc I (H-3)
5.91 C’	169.5	5.09 Glc II (H-2)	5.89 C’	168.1	5.08 Glc II (H-2)
7.12 E	165.7	5.92 Glc I (H-4)			
			6.95 A’	164.3	6.24 Glc II (H-1)

Methanol *d*_4_. ^a 1^H-NMR 500 MHz, ^13^C-NMR 126 MHz, ^b 1^H-NMR 600 MHz, ^13^C-NMR 200 MHz.

**Table 4:** ^13^C and ^1^H-NMR data of 3 ^a^.

	^1^H	^13^C	^1^H	^13^C
Glucose			Xylose	
1	4.35 d (*J* = 7.8)	102.5	4.34 d (*J* = 7.6)	102.5
2	3.21 dd (*J* = 7.8, 9.1)	73.8	3.21 dd (*J* = 7.6, 9.1)	74.3
3	3.41 dd (*J* = 6.5, 9.1)	77.1	3.36 ft (*J* = 8.8)	75.8
4	3.39 dd (*J* = 6.5, 8.1)	76.6	3.49 dd (*J* = 4.8, 8.5)	70.2
5	3.43 dd (*J* = 8.1 10.4)	70.1	3.70 dd (*J* = 4.8, 11.5)	68.7
			3.98 dd (*J* = 2.1, 11.5)	
6	3.18 dd (*J* = 10.1, 11.4)	65.9		
	3.81 dd (*J* = 5.3, 11.4)			
Aglicone				
1a	5.18 dd (*J* = 3.6, 17.3)	116.1		
1b	5.05 dd (*J* = 3.6, 5.5)			
2	5.82 ddd (*J* = 7.1, 10.5, 17.3)	140.2		
3	4.09 q (*J* = 5.5, 8.7)	82.1		
4a	1.45 d (*J* = 8.7)	34.9		
4b	1.60 dd (*J* = 10, 13.6)			
5	1.22 d (*J* = 11, 13.6)	32.2		
6	1.22 d (*J* = 11)	24.9		
7	1.30 ft (*J* = 11, 7.0)	22.9		
8	0.8 ft (*J* = 7.2)	14.1		

^a^ Acetone *d*_6_ + D_2_O. ^1^H-NMR 400 MHz, ^13^C-NMR 100 MHz.

## Supplementary Materials

The following are available online, Figures S1–S19: 1D and 2D NMR spectra, Circular Dichroism spectra and High Resolution Mass spectra of merianin A (**1**), merianin B (**2**) and **3**.
