# Supplementary material for: Hydrolysable Tannins and Biological Activities of Meriania hernandoi and Meriania nobilis (Melastomataceae)
Source: Molecules. 2019 Feb 19;24(4):746. doi: 10.3390/molecules24040746 (PMC6412690; doi:10.3390/molecules24040746)
Supplement: Supplementary file 1 [file molecules-24-00746-s001.pdf]

Supplementary Material

Hydrolysable Tannins and Biological Activities of *Meriania hernandoi* and *M. nobilis* (Melastomataceae)

Claudia Lorena Valverde Malaver [1], Ana Julia Colmenares Dulcey [1], Rosa María Varela Montoya [2], José María Gonzalez Molinillo [2], Francisco Macías [2] and José Hipólito Isaza Martínez [1,*]

[1]Universidad del Valle, Faculty of Natural and Exact Sciences, Department of Chemistry. GIPNA. Cali, Colombia 1; claudia.valverde@correounivalle.edu.co, ana.colmenares@correounivalle.edu.co,
*****Correspondence: jose.isaza@correounivalle.edu.co; Tel.: +57-2-321-2125

[2]Universidad de Cadiz, School of Sciences, Department of Organic Chemistry. Instituto de Biomoléculas (INBIO). Cadiz, Spain;

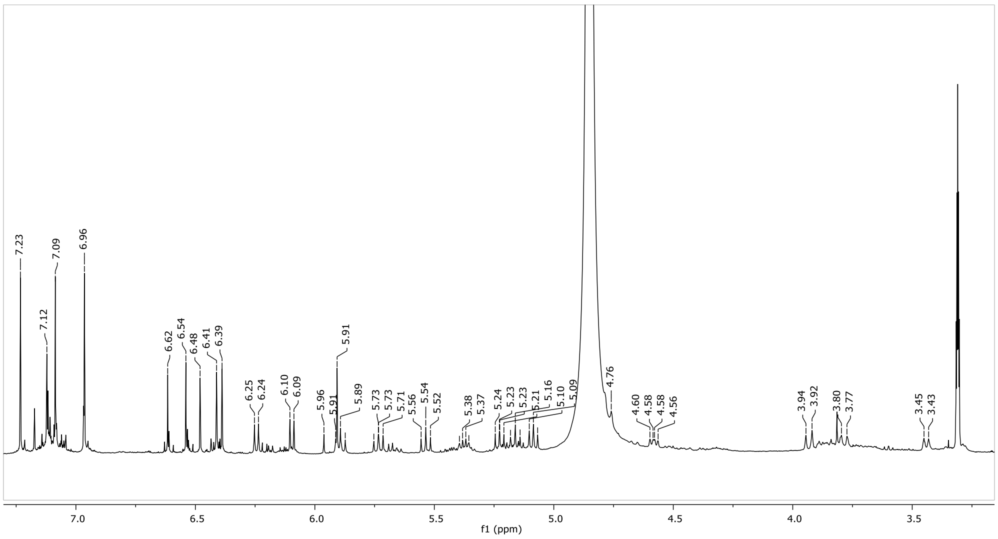

Figure S1: ^{1}H NMR spectrum (500 MHz, Methanol *d₄*) of Merianin A (**1**)

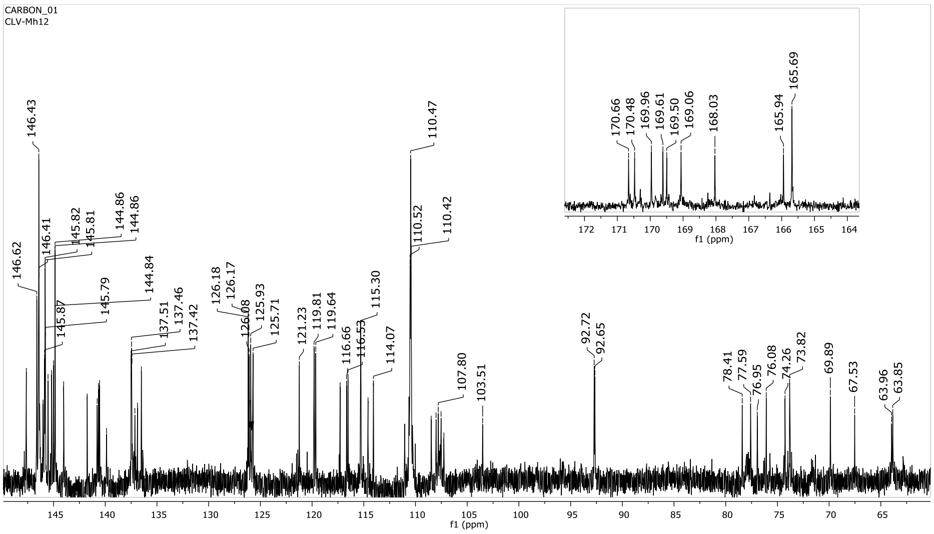

Figure S2: ^{13}C NMR spectrum (125 MHz, Methanol *d₄*) of merianin A (**1**)

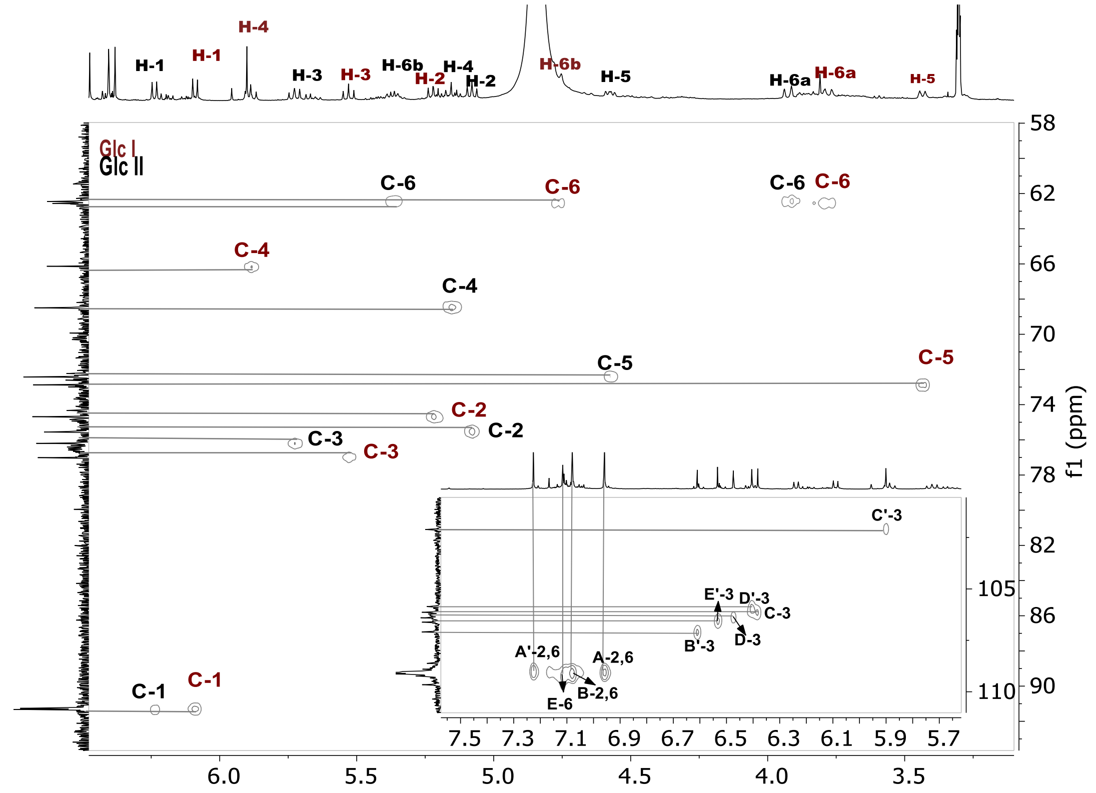

Figure S3: HSQC spectrum of merianin A (**1**).

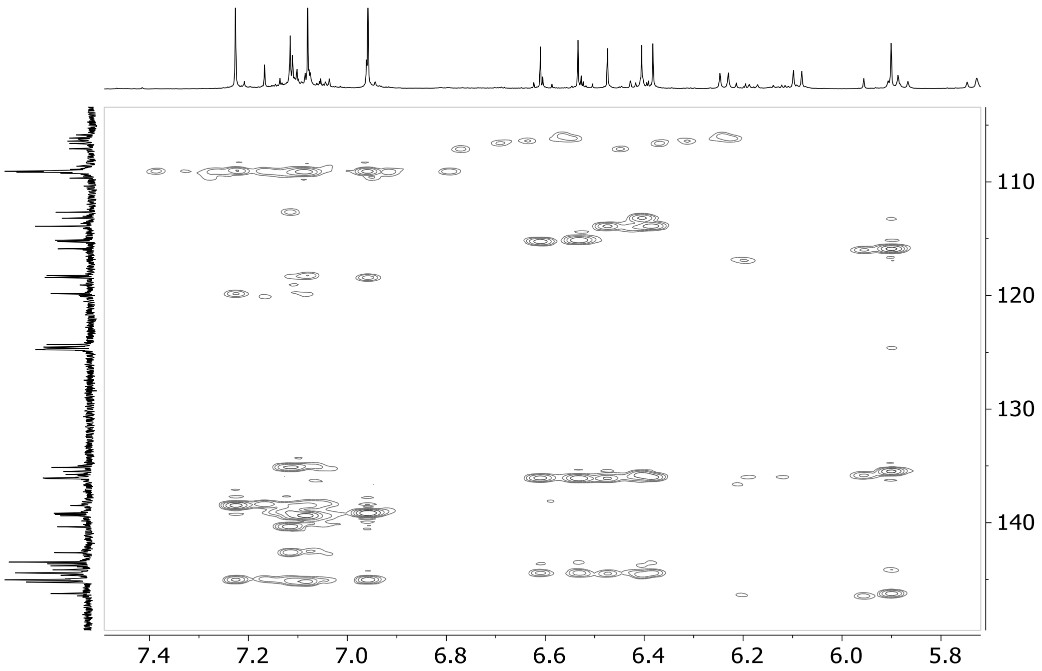

Figure S4: HMBC spectrum of merianin A (**1**).

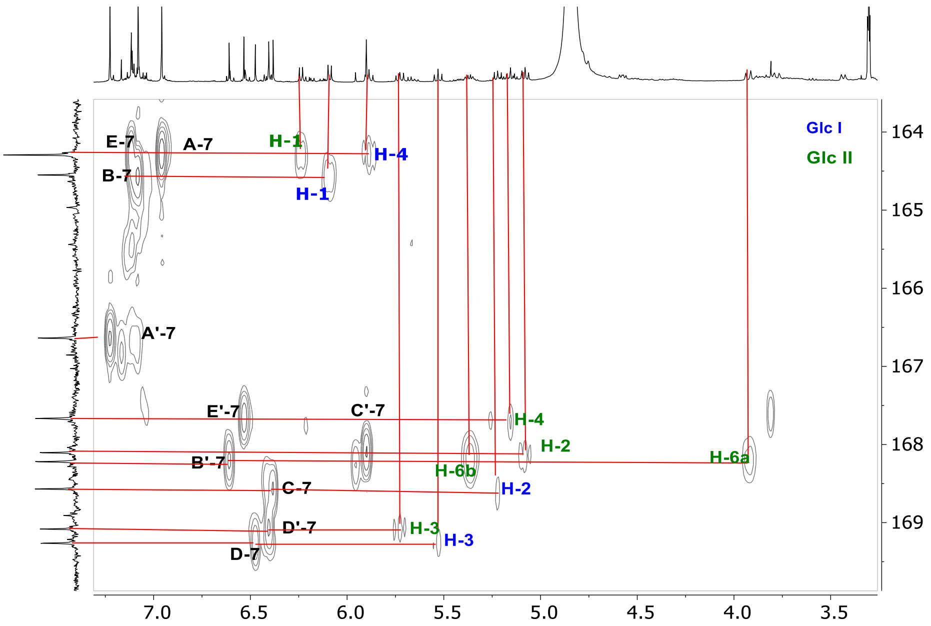

Figure S5. Connectivity's between aromatic and glucose protons through ester carbonyl carbon (3 bond coupling, expansion of HMBC) of merianin A (**1**)

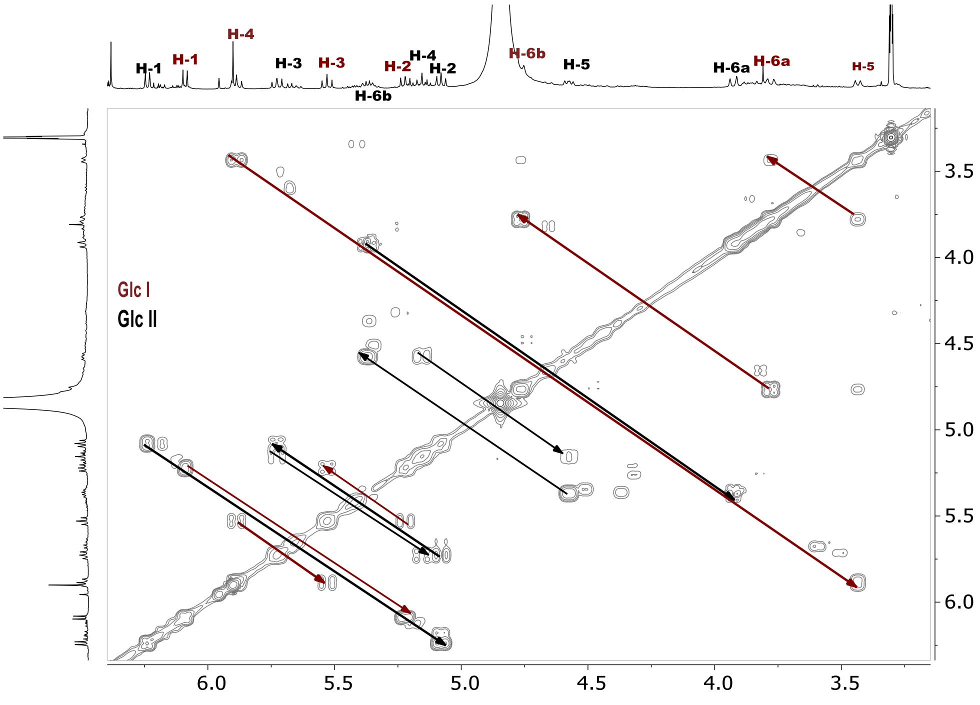

Figure S6: ^{1}H-^{1}HCOSY spectrum of merianin A (**1**).

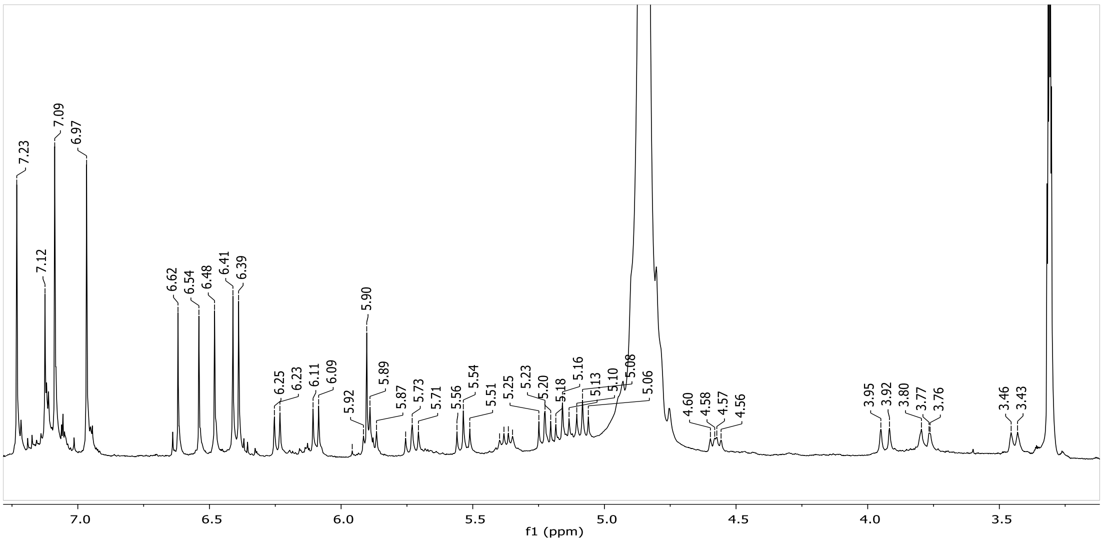

Figure S7: ^{1}H NMR spectrum (600 MHz, Methanol d_4) of merianin B (**2**)

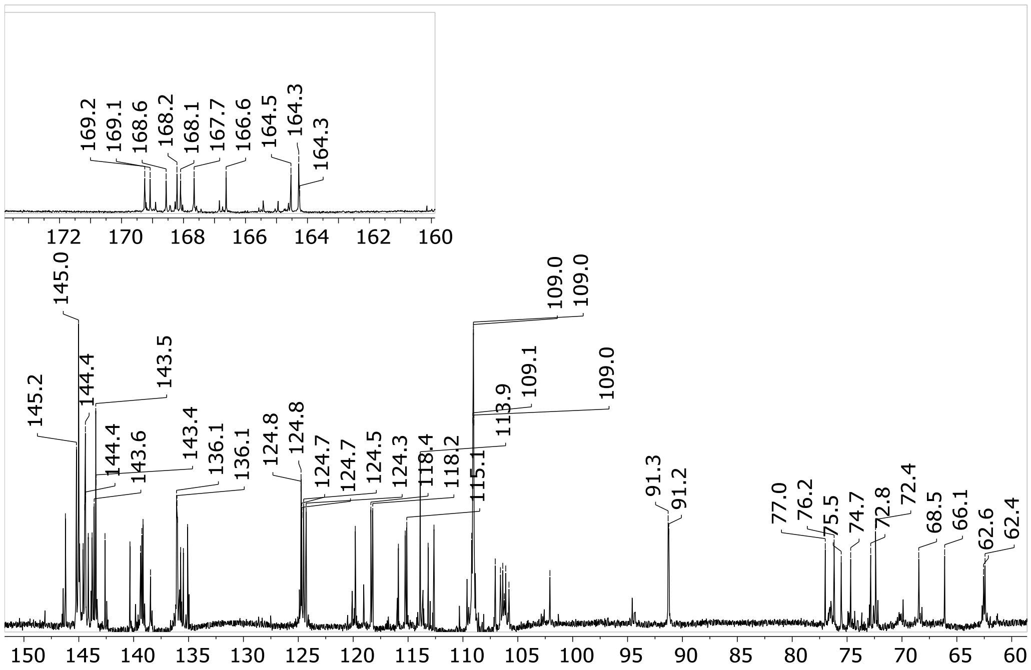

Figure S8: ^{13}C NMR spectrum (200 MHz, Methanol d_4) of merianin B (**2**)

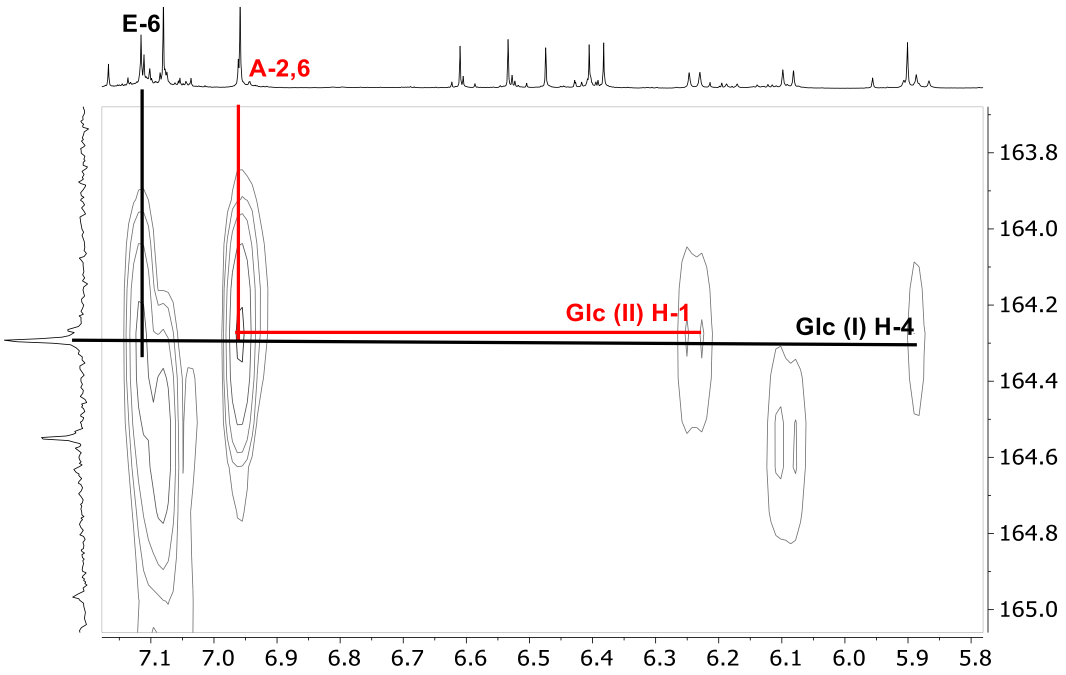

Figure S9: correlation of carbonyl group and glucose to ^{3}J (expansion of HMBC) of merianin A (**1**)

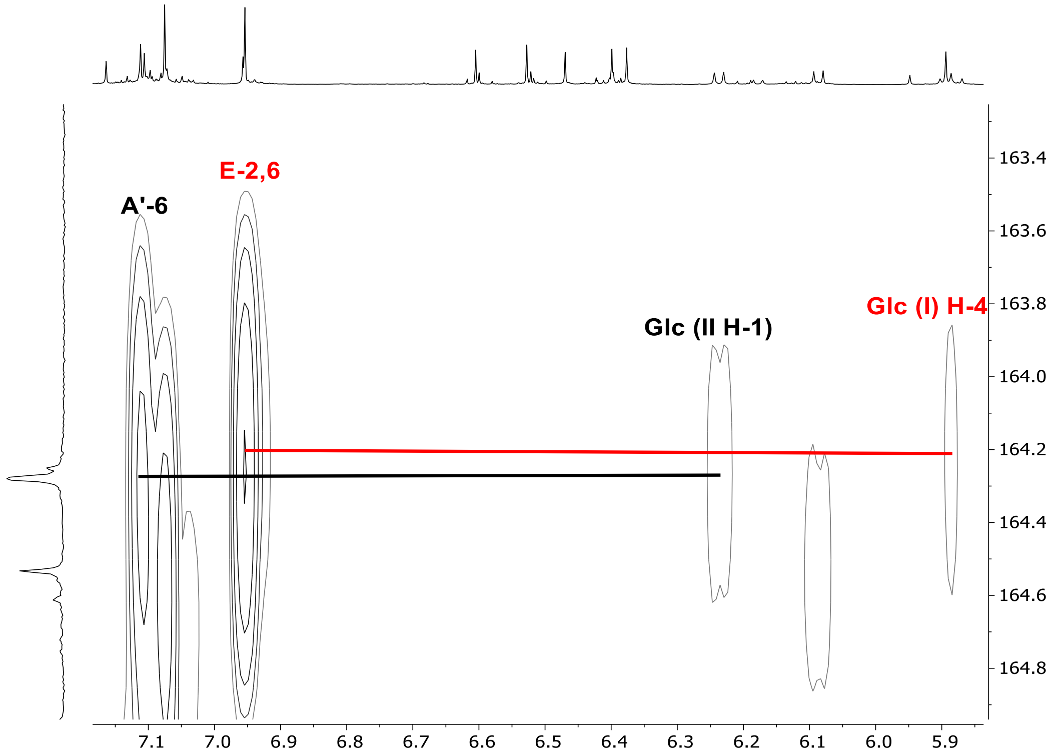

Figure S10: correlation of carbonyl group and glucose to ^{3}J (expansion of HMBC) merianin B (**2**).

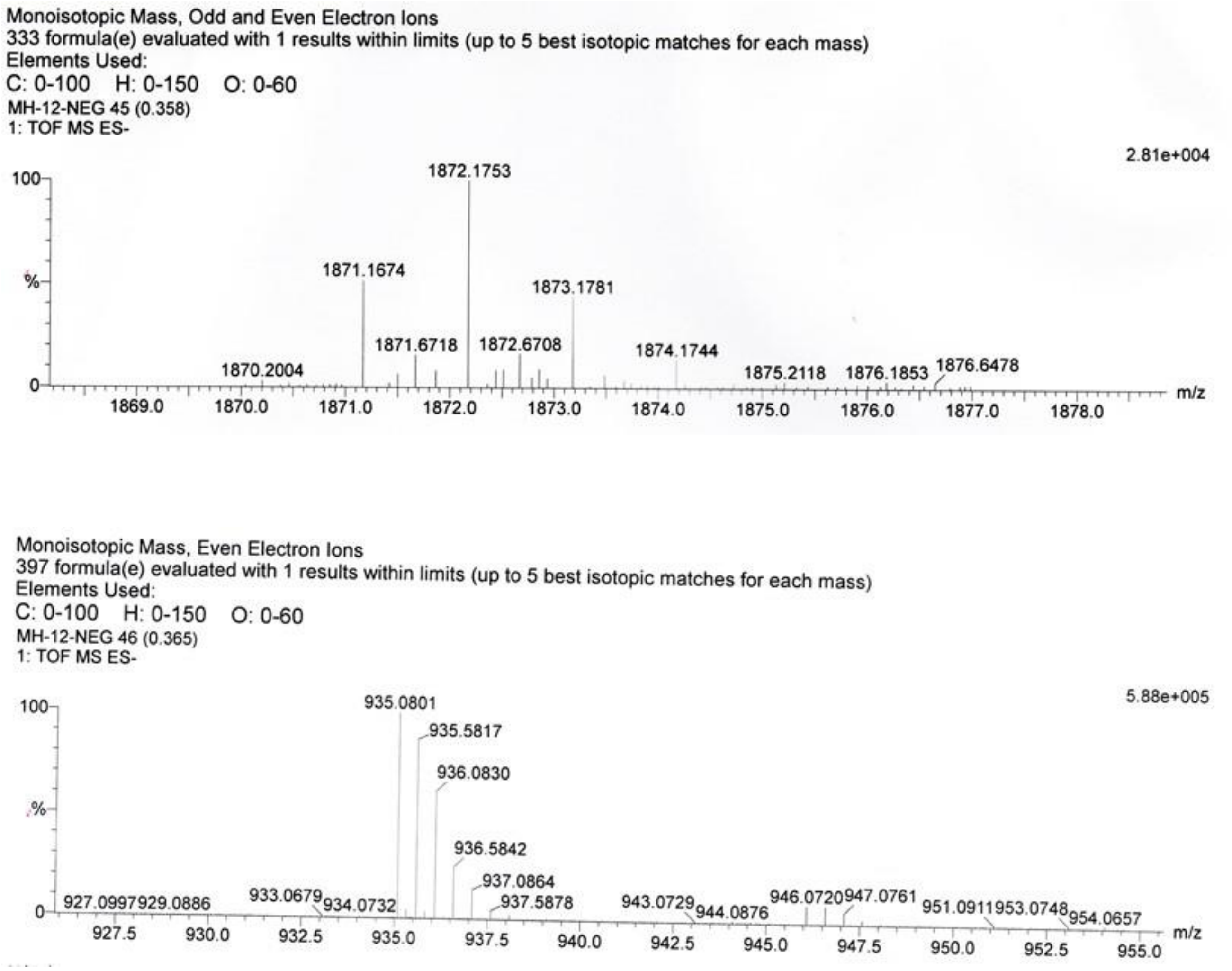

Figure S11: ESI-MS spectrum of merianin A (**1**). (a) Pseudo-molecular ion peak at *m/z* 1871.1674 $[M-H]^-$ in negative mode, (b) doubly charged pseudo-molecular ion peak at *m/z* 935.0801 $[M-2H]^{2-}$ in negative mode.

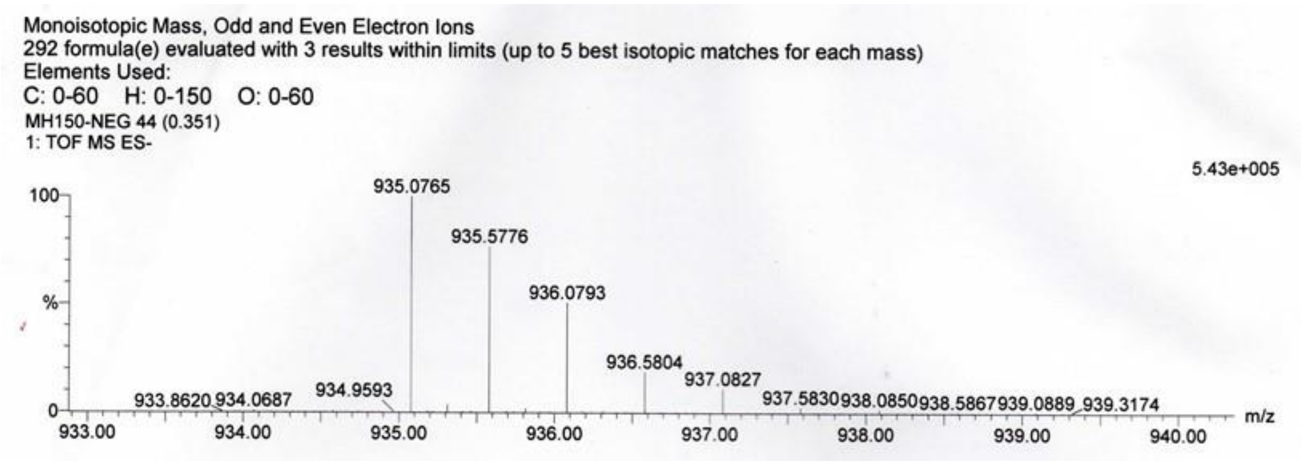

Figure S12: ESI-MS spectrum of merianin B (**2**). Spectrum show a doubly charged pseudo-molecular ion peak at *m/z* 935.0801 $[M-2H]^{2-}$ in negative mode.

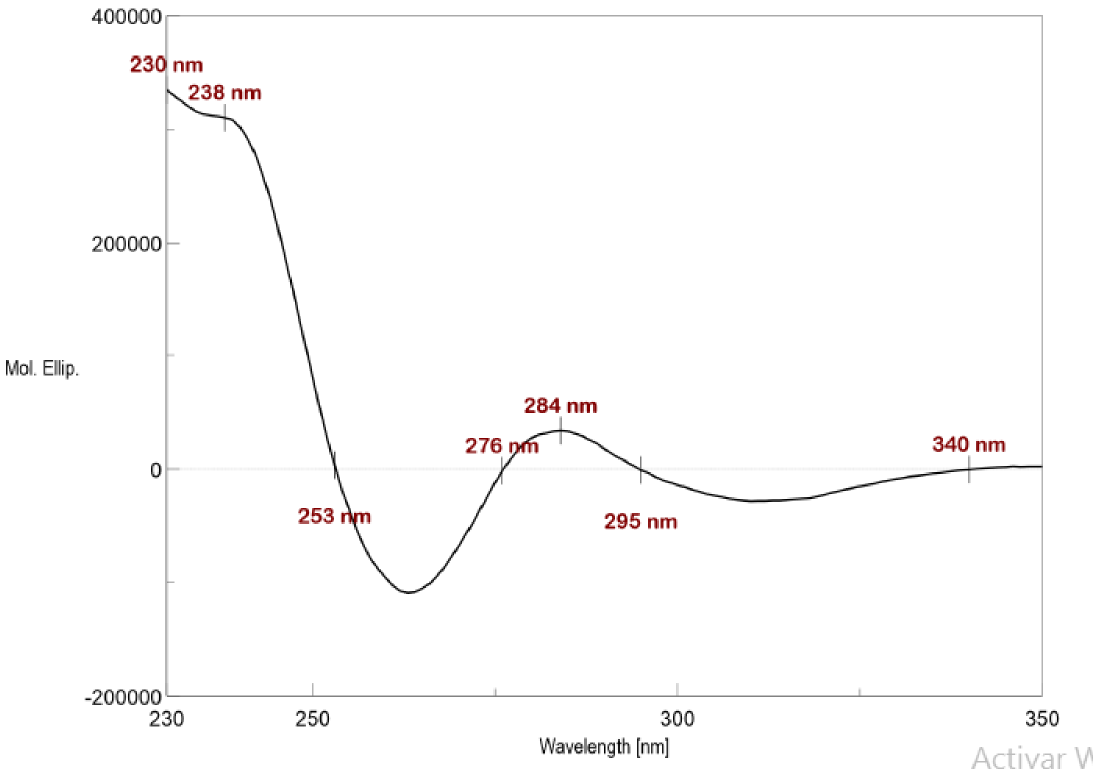

Figure S13: CD Spectrum (MeOH) of merianin A (**1**).

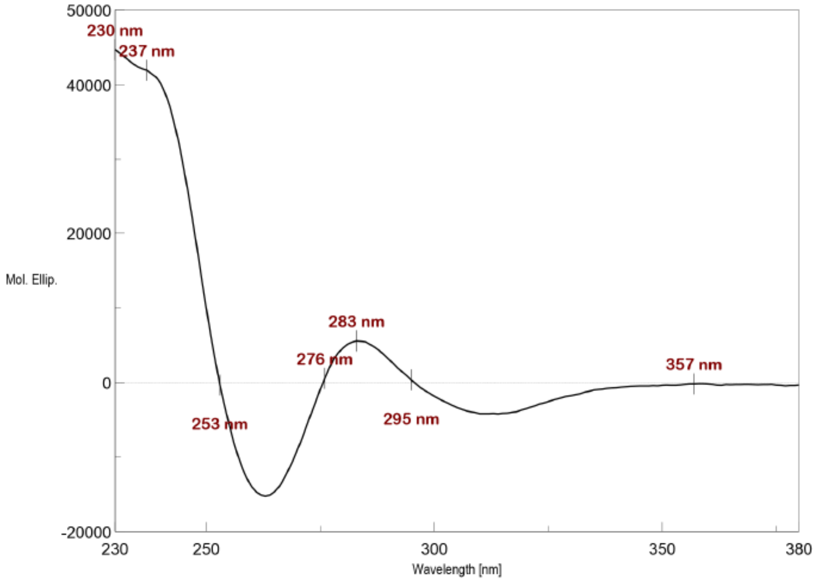

Figure S14: CD Spectrum (MeOH) of merianin B (**2**).

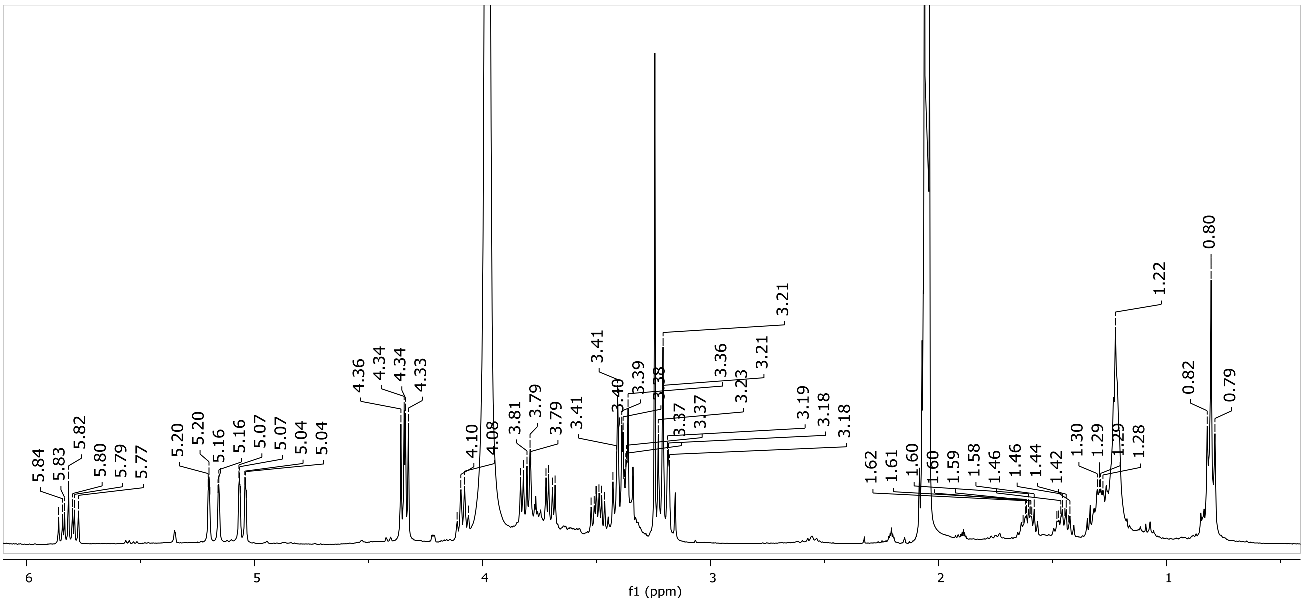

Figure S15 ^{1}H NMR spectrum (400 MHz, Methanol d_4) of **3.**

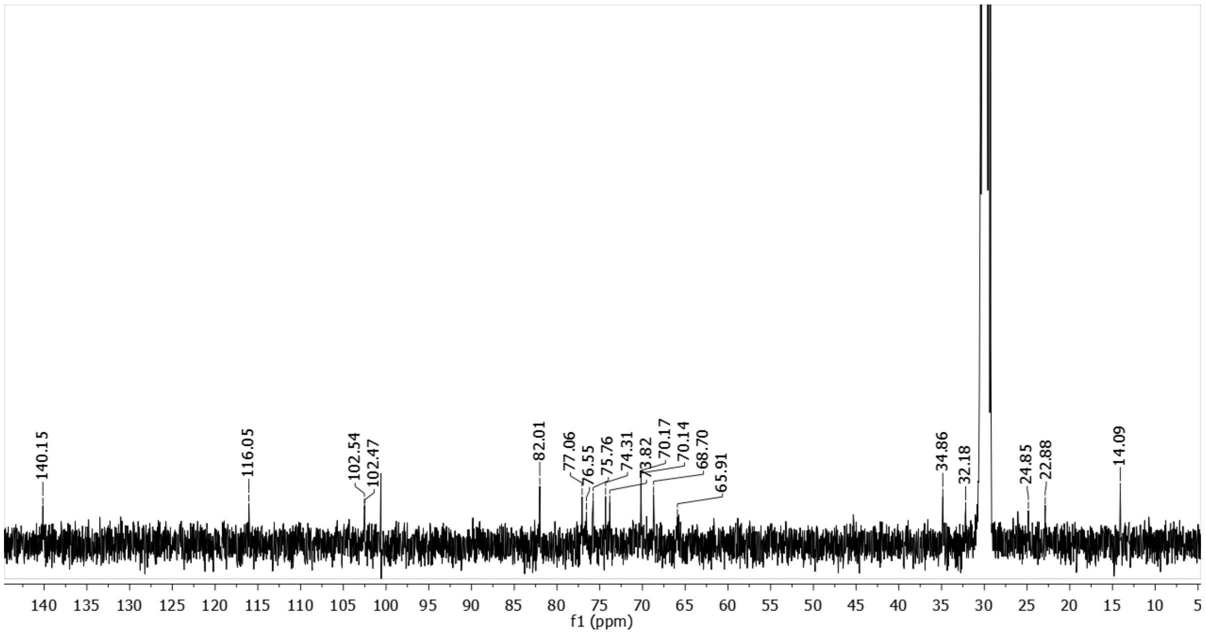

Figure S16: ^{12}C NMR spectrum (100 MHz, Methanol d_4) of **3.**

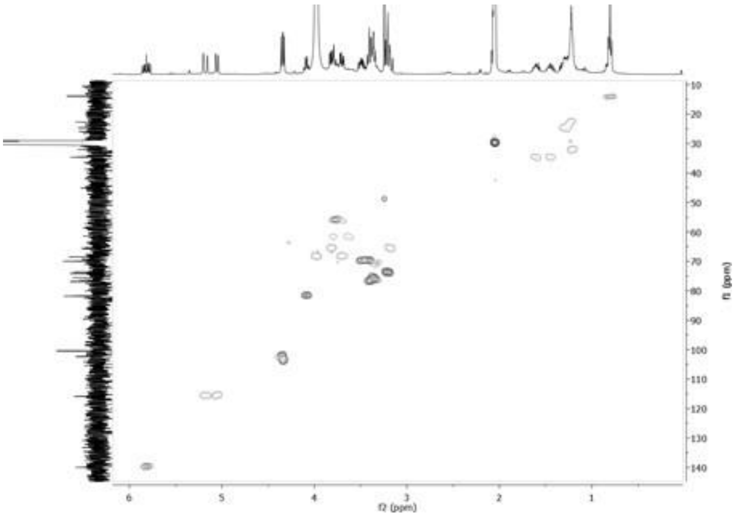

Figure S17: HSQC spectrum of **3.**

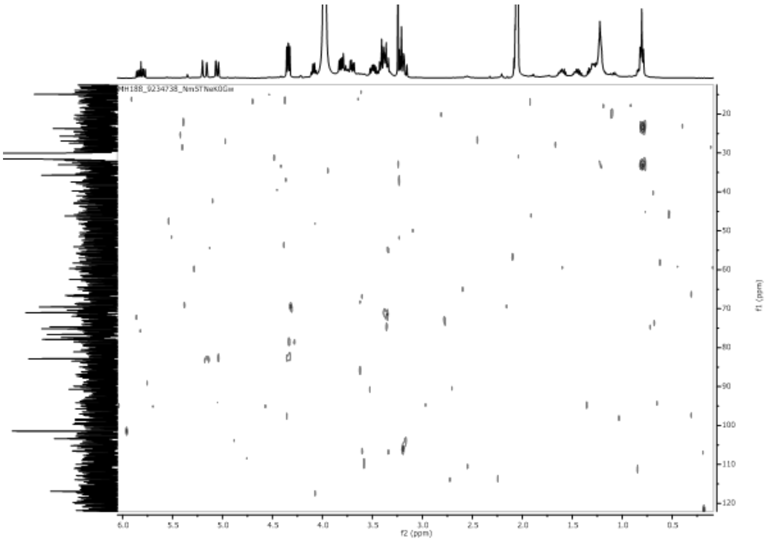

Figure S18: HMBC spectrum of **3**

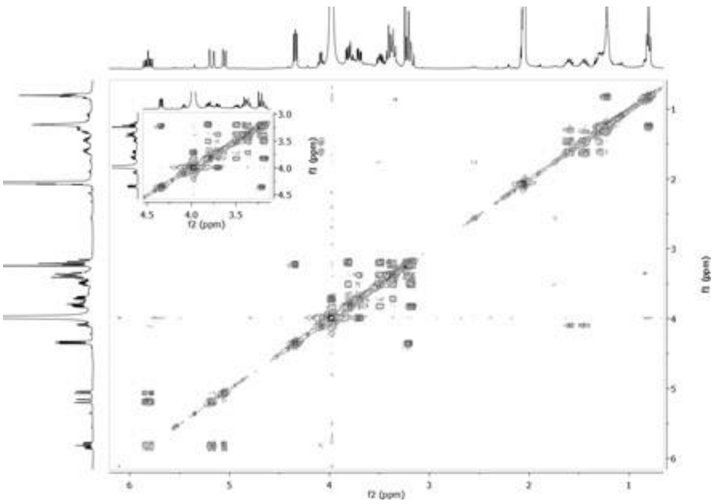

Figure S19: ^{1}H-^{1}HCOSY spectrum of **3**.

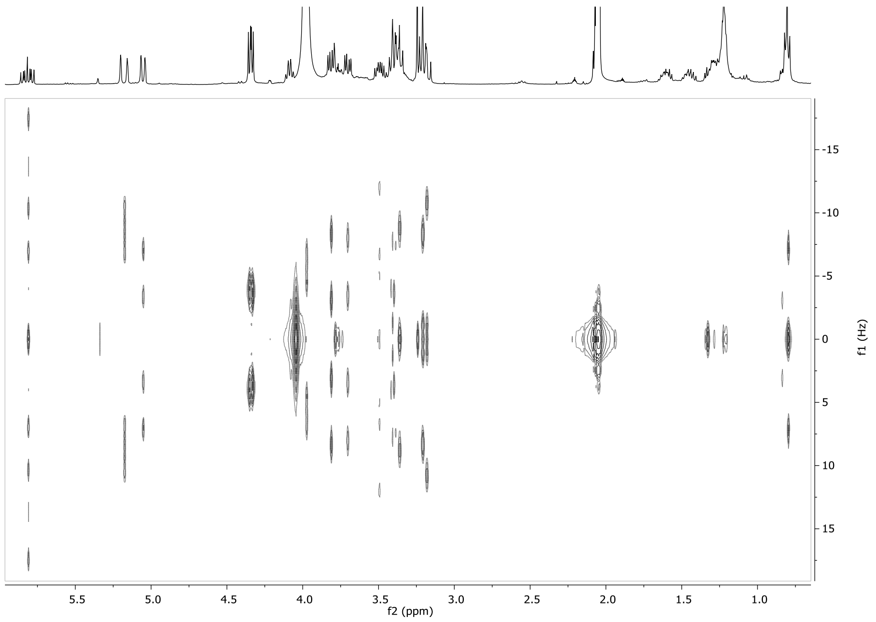

Figure S20: JRES spectrum of **3**

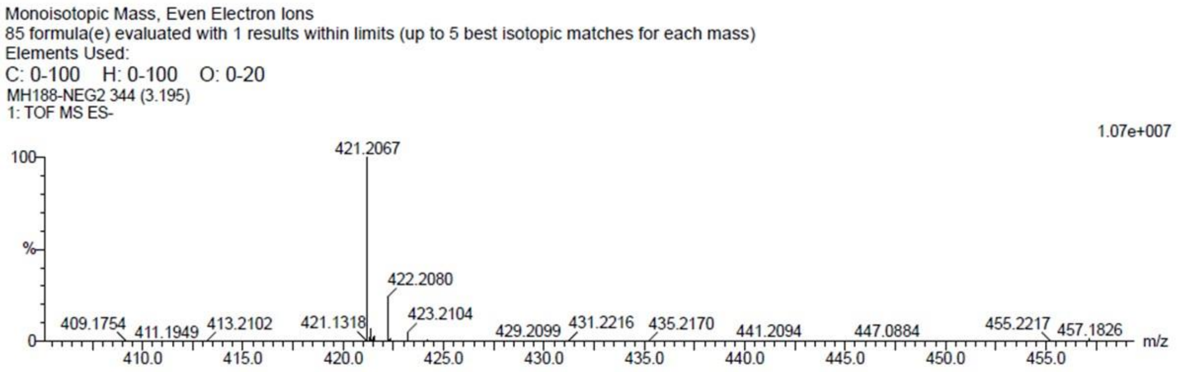

Figure S21: ESI-MS spectrum of **3**. Pseudo-molecular ion peak at *m/z* 421.2067 $[M-H]^-$ in negative mode.
